# Inducible Deletion of CD28 Prior to Secondary *Nippostrongylus brasiliensis* Infection Impairs Worm Expulsion and Recall of Protective Memory CD4^+^ T Cell Responses

**DOI:** 10.1371/journal.ppat.1003906

**Published:** 2014-02-06

**Authors:** Hlumani Ndlovu, Mathew Darby, Monika Froelich, William Horsnell, Fred Lühder, Thomas Hünig, Frank Brombacher

**Affiliations:** 1 International Center for Genetic Engineering and Biotechnology (ICGEB), Cape Town Component and Institute of Infectious Diseases and Molecular Medicine (IIDMM), Division of Immunology, University of Cape Town, Cape Town, South Africa; 2 Institute for Virology and Immunology, University of Würzburg, Würzburg, Germany; 3 Department of Neuroimmunology, Institute of Multiple Sclerosis Research and The Hertie Foundation, University Medical Center Göttingen, Göttingen, Germany; New York University, United States of America

## Abstract

IL-13 driven Th2 immunity is indispensable for host protection against infection with the gastrointestinal nematode *Nippostronglus brasiliensis*. Disruption of CD28 mediated costimulation impairs development of adequate Th2 immunity, showing an importance for CD28 during the initiation of an immune response against this pathogen. In this study, we used global CD28^−/−^ mice and a recently established mouse model that allows for inducible deletion of the *cd28* gene by oral administration of tamoxifen (CD28^−/lox^Cre^+/−^+TM) to resolve the controversy surrounding the requirement of CD28 costimulation for recall of protective memory responses against pathogenic infections. Following primary infection with *N. brasiliensis*, CD28^−/−^ mice had delayed expulsion of adult worms in the small intestine compared to wild-type C57BL/6 mice that cleared the infection by day 9 post-infection. Delayed expulsion was associated with reduced production of IL-13 and reduced serum levels of antigen specific IgG1 and total IgE. Interestingly, abrogation of CD28 costimulation in CD28^−/lox^Cre^+/−^ mice by oral administration of tamoxifen prior to secondary infection with *N. brasiliensis* resulted in impaired worm expulsion, similarly to infected CD28^−/−^ mice. This was associated with reduced production of the Th2 cytokines IL-13 and IL-4, diminished serum titres of antigen specific IgG1 and total IgE and a reduced CXCR5^+^ T_FH_ cell population. Furthermore, total number of CD4^+^ T cells and B220^+^ B cells secreting Th1 and Th2 cytokines were significantly reduced in CD28^−/−^ mice and tamoxifen treated CD28^−/lox^Cre^+/−^ mice compared to C57BL/6 mice. Importantly, interfering with CD28 costimulatory signalling before re-infection impaired the recruitment and/or expansion of central and effector memory CD4^+^ T cells and follicular B cells to the draining lymph node of tamoxifen treated CD28^−/lox^Cre^+/−^ mice. Therefore, it can be concluded that CD28 costimulation is essential for conferring host protection during secondary *N. brasiliensis* infection.

## Author Summary

CD28 is an important costimulatory molecule, involved in the activation of naive T cells, enhancing cytokine production, preventing T cell anergy and apoptosis. Furthermore, CD28 plays a crucial role in the organisation of secondary lymphoid tissue by assisting in the recruitment of T cells into the B cell follicles, thus promoting germinal center formation, isotype switching and B cell maturation. The requirement of CD28 costimulatory signalling during recall of memory responses against infections has remained controversial. Hence, here we utilised a mouse model that allowed for inducible deletion of the *cd28* gene (CD28^−/lox^Cre^+/−^) by oral administration of tamoxifen to resolve this controversy. CD28^−/−^ mice and mice given tamoxifen prior to secondary infection failed to expel adult *N. brasiliensis* worms. This was related to reduced production of the Th2 cytokines IL-13 and IL-4, diminished type 2 antibody titres, and a reduced number of memory CD4^+^ T cells. In summary, CD28 is crucial for protection against *N. brasiliensis* secondary infection and plays a key role in the recruitment of T_FH_ cells, memory CD4^+^ T cells and follicular B cells.

## Introduction

CD28 is considered to be the main co-stimulator of T cells, providing a critical signal for activation of naive T cells [1], [2], [3]. Interactions between CD28 and its ligands CD80/CD86 enhances cytokine production, prevents T cell anergy and protects against apoptosis [4], [5]. These CD28 dependent interactions are important during the initiation of T cell mediated immunity against a number of infections. Mice deficient in CD28 failed to develop adequate Th2 immune response during infection with *S. mansoni* [6], *L. major* [7] and *N. brasiliensis* [8], [9]. In contrast, infection of CD28^−/−^ mice with *H. polygyrus* did not hamper normal development of Th2 immune response [10].

The absence of CD28 alters the organisation of secondary lymphoid tissue by affecting recruitment of T cells to B cell follicles, impairing germinal centre development [11], [12], [13], isotype switching, B cell maturation and development of memory B cells. This is linked to diminished recruitment of CXCR5^+^ T_FH_ cells which localise within the B cell follicles [14], [15], [16], [17]. T_FH_ cells produce IL-21, a key cytokine involved in isotype switching and differentiation of plasma cells [15]. CD28^−/−^ mice infected with *S. mansoni* [6] or *Leishmania major* [7] failed to produce antigen specific type 2 antibodies IgG1 and IgE. Taken together this demonstrates an important role for CD28 in co-ordinating B cell responses.

Studies suggest that CD28 is not required during recall of memory T cell responses to infection with *N. brasiliensis* [8] and *H. polygyrus* [18]. Furthermore, infection of CD28^−/−^ mice with fungi *B. dermatitidis* revealed maintenance of memory T cells is CD28 independent [19]. In fact, some studies suggested that recall of memory responses may be dependent on other co-stimulatory molecules such as inducible costimulator (ICOS) or 4-1BB [20], [21], [22]. In contrast, development of effector and memory CD4^+^ T cells was reduced in the absence of CD28 during *T. gondii* infection [23]. Recall of memory responses to persistent viral infections is dependent on CD28 [24], [25]. Therefore, the importance of CD28 during development and recall of memory responses remains controversial. There have been attempts to address this issue by blocking CD80 and CD86 or by transfer of memory T cells into CD80/CD86 deficient mice [26]. However, both approaches deprive CTLA-4 (CD152) of its ligands thus caution must be exercised when interpreting these data. Hence, new approaches that don't suffer from these additional effects are required to solve the conundrum surrounding the contribution of CD28 during recall of memory responses to infections.

Infection of mice with *Nippostrongylus brasiliensis* triggers a host protective immune response characterised by increased production of Th2 cytokines IL-13 and IL-4 [27], [28], [29], goblet cell hyperplasia [30] eosinophilia [31] and elevated levels of serum IgG1 and IgE [28], [32]. Infection with *N. brasiliensis* begins when third stage larvae (L3) penetrate the skin and migrate, via the circulatory system, into the lungs. Larvae enter the airways, from which they are coughed up and swallowed. The larvae mature into adult worms that produce eggs upon reaching the small intestinal lumen. Immune-competent BALB/c mice clear *N. brasiliensis* infection after approximately 9 days post-infection [33]. Secondary infection with *N. brasiliensis* induces a potent memory immune response that results in poor worm maturation and inhibits egg production [34].

Our understanding of mechanisms mediating recall of protective immunity to *N. brasiliensis* infection is continuously expanding. Studies conducted in mice defective of eosinophilopoeisis showed the importance of eosinophils in driving recall of protective immunity to *N. brasiliensis* re-infection [35], [36], [37]. Furthermore, recent studies have demonstrated an essential role of lung-resident CD4^+^ T cells in mediating host resistance to *N. brasiliensis* re-infection [38], [39], and this was shown to be dependent on IL-4Rα signalling [39]. Earlier studies conducted in B-cell deficient mice suggested that B cells do not play a role in the resolution *N. brasiliensis* secondary infection [40]. However, a recent study by us showed that interfering with IL-4Rα signalling specifically on B cells impaired worm expulsion during secondary challenge with *N. brasiliensis*, suggesting that IL-4/IL-13 responsive B cells are required for recall of protective immunity [41]. This was shown to be related to impaired IL-13 production by B cells and reduced expression of markers associated with antigen presentation [41].

The aim of this study was to evaluate the importance of CD28 in initiating protective Th2 immunity against both primary and secondary infections with *N. brasiliensis*. Our findings demonstrate that CD28 is required for initiation of protective Th2 immunity against primary infection with *N. brasiliensis*. Furthermore, the absence of CD28 impairs development of memory CD4^+^ T cell responses resulting in failure to clear adult *N. brasiliensis* worms during secondary infection. Failure to resolve infection was associated with reduced production of Th2 cytokines, particularly IL-13 and IL-4, abrogated humoral immunity and failure to expand the memory CD4^+^ T cell compartment.

## Results

### CD28 costimulatory signalling is required for optimal immunity against *N. brasiliensis* infection

**Figure 1:**
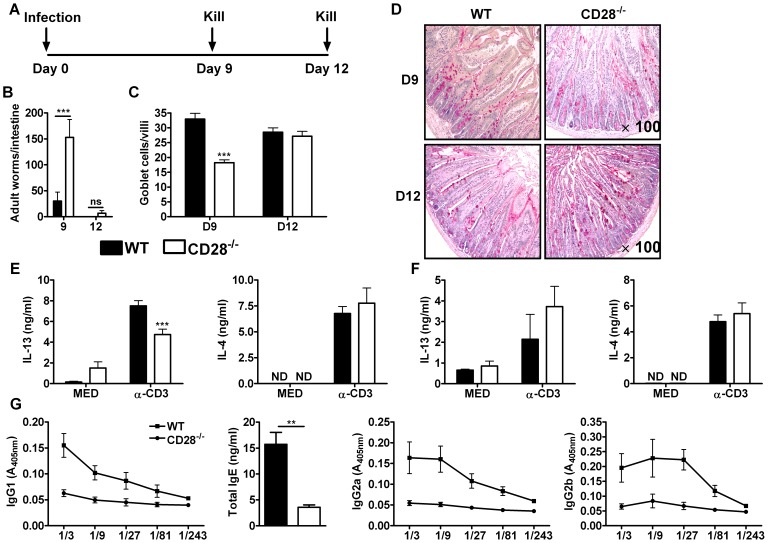
Delayed worm expulsion in CD28 deficient mice during primary *N. brasiliensis* infection. Wild-type (C57BL/6) and CD28^−/−^ mice were infected with 500 L3 *N. brasiliensis* and killed 9 and 12 days post-infection. (**A**), Schematic showing experimental set-up. (**B**), Intestinal worm burdens were quantified. (**C**), Goblet cells per villi were enumerated. (**D**), PAS staining of mucus producing goblet cells in the intestinal tissue. Cytokine production by total mesenteric lymph node cells collected 9 days (**E**) or 12 days (**F**) post-infection was determine by ELISA after re-stimulation of total cells in media or with 20 µg/ml α-CD3 for 72 h at 37°C. (**G**), Serum antibody titres of *N. brasiliensis* specific IgG1, IgG2a, IgG2b and total IgE were determined by ELISA. Data are representative of two independent experiments. n = 4–6 mice per group. *P<0.05, **P<0.01, and ***P<0.001 vs C75BL/6 mice using two-tailed Mann-Whitney non-parametric Student's *t*-test or Two-Way ANOVA with Bonferroni's post test.

In order to investigate whether CD28 is required for the development of protective immunity against *N. brasiliensis* infection, CD28^−/−^ mice were infected with 500 infective *N. brasiliensis* larvae and killed 9 and 12 days post-infection (Fig. 1A). Infected CD28^−/−^ mice had significantly higher intestinal adult worm burdens at day 9 post-infection compared to infected wild-type C57BL/6 mice (Fig. 1B). At day 12 post-infection, infected C57BL/6 mice cleared the worms as well as most of the infected CD28^−/−^ mice (Fig. 1B). Associated with transiently increased worm burdens in infected CD28^−/−^ mice was the number of mucus producing goblet cells that were reduced at day 9 post-infection in this mouse strain compared to control mice (Fig. 1C). Histological examination of periodic-acid Schiff (PAS) stained intestinal sections further confirmed that infected CD28^−/−^ mice had impaired goblet cell hyperplasia at day 9 post-infection compared to infected C57BL/6 mice (Fig. 1C). Mucus production by goblet cells is crucial for mediating expulsion of adult *N. brasiliensis* worms [28], [42], [43], [44].

Production of Th2 cytokines, particularly IL-13, drives the development of host protective immunity to *N. brasiliensis* [28]. To investigate the impact of CD28 deficiency in the production of host protective Th2 cytokines, cytokine production by total mesenteric lymph node (MLN) cells cultured in media or in the presence of 20 µg/ml α-CD3 was determined by sandwich ELISA. Total MLN cells re-stimulated with α-CD3 resulted in significantly reduced production of IL-13 by CD28^−/−^ mice at day 9 post-infection (Fig. 1E) and was similar at day 12 (Fig. 1F) compared to wild-type mice, a cytokine crucial for worm clearance [27], [28], [39], [42], [45]. Production of IL-4 was unaltered between the two groups (Fig. 1E and F). Little or no cytokine production was detected in media control supernatants from all groups of mice (Fig. 1E and F). Finally, serum titers of both type 1 (IgG2a and IgG2b) and type 2 (IgG1 and total IgE) antibodies were markedly reduced in CD28^−/−^ mice compared to C57BL/6 mice (Fig. 1G), suggesting that CD28 deficiency results in a general attenuation of the humoral immune response during *N. brasiliensis* infection. Together, these data demonstrated that CD28 is required for optimal immunity against *N. brasiliensis* primary infection.

### CD28 is required for protective memory responses to *N. brasiliensis* infection

In order to investigate the contribution of CD28 in host protective memory responses, we took advantage of inducible CD28 deleting mouse strain CD28^−/lox^, rosa (CreER^T2^), here referred to (CD28^−/lox^Cre^+/−^), a suitable experimental model system, recently generated by us [46], [47]. CD28^−/lox^ mice (exon 2 and 3 are flanked by *lox*P) were intercrossed with rosaCreER^T2^ mice to generate CD28^−/lox^, rosa (CreER^T2^) mice. Oral administration of estrogen analogue tamoxifen allowed for translocation of estrogen-receptor-Cre fusion protein into the nucleus, where Cre recombinase carried out efficient deletion of the *cd28* gene [46], [47], [48].

**Figure 2:**
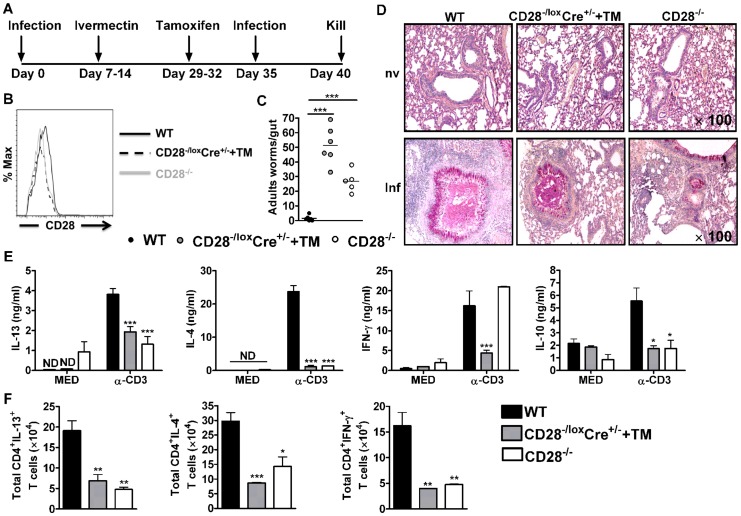
CD28 is required for development of protective recall responses to re-infection with *N. brasiliensis*. Wild-type (C57BL/6), tamoxifen treated CD28^−/lox^Cre^+/−^ and CD28^−/−^ mice were infected with 500 L3 *N. brasiliensis*, treated with Ivermectin, then re-infected with 500 L3 *N. brasiliensis* at day 35 and killed 5 days post-infection. (**A**), Schematic showing experimental set-up. (**B**), Histogram showing the efficiency of CD28 deletion on CD4^+^ T cells. (**C**), Intestinal worm burdens were quantified. (**D**), PAS staining of pulmonary mucus producing goblet cells from naive and infected mice (**E**), Cytokine production by total mediastinal lymph node cell re-stimulated in media or in the presence of 20 µg/ml α-CD3 was determined by ELISA. (**F**), Total number of CD4^+^ T cells producing IL-13, IL-4 and IFN-γ after restimulation of total MST cells with 50 ng/ml PMA, 250 ng/ml ionomycin and 200 µM monensin *in vitro*. Data is representative of two independent experiments. n = 5–6 mice per group. *P<0.05, **P<0.01, and ***P<0.001 vs C57BL/6 using One-Way or 2-Way ANOVA with Bonferroni's post test.

C57BL/6, CD28^−/lox^Cre^+/−^ and CD28^−/−^ mice were infected with 500 infective *N. brasiliensis* larvae, treated with Ivermectin and rested for 21 days (Fig. 2A). At day 29, CD28^−/lox^Cre^+/−^ mice were treated with tamoxifen in vegetable oil (CD28^−/lox^Cre^+/−^+TM) for four consecutive days (Fig. 2A). At day 35, mice were re-infected with 500 L3 *N. brasiliensis* and killed 5 days post-infection (Fig. 2A). Efficient deletion of CD28 was confirmed by flow cytometric analysis of CD4^+^ T cells. Here, oral administration of tamoxifen in CD28^−/lox^Cre^+/−^ mice resulted in similar levels of CD28 expression as was found in infected CD28^−/−^ mice (Fig. 2B, Fig. S1A). Since eosinophils are important mediators of anti-helminth immunity and activated human eosinophils were shown to express CD28 [35], [36], [49], we tested for CD28 surface expression by this cell type in infected mice. Mouse eosinophils were negative in CD28 surface expression, as shown by flow cytometric analysis of CD28 expression by eosinophils from CD28^−/−^ mice and C57BL/6 mice (data not shown). CD28^−/lox^Cre^+/−^ mice treated with tamoxifen prior to secondary challenge with *N. brasiliensis* adult worms exhibited high intestinal worm burdens similar to CD28^−/−^ mice (Fig. 2C). Both infected tamoxifen treated CD28^−/lox^Cre^+/−^ mice and CD28^−/−^ mice had significantly higher worm burdens than infected wild-type C57BL/6 mice (Fig. 2C) or infected untreated CD28^−/lox^Cre^+/−^ littermate control mice (Fig. S1B). Goblet cell hyperplasia was similar between wild-type C57BL/6 mice and tamoxifen treated CD28^−/lox^Cre^+/−^ mice (Fig. 2D). In contrast, CD28^−/−^ mice showed reduced goblet cell hyperplasia indicated by reduced mucus producing cells compared to wild-type control mice (Fig. 2D). Therefore, these data suggest that CD28 costimulation is essential for host protection during *N. brasiliensis* secondary infection.

To investigate the mechanism resulting in impaired protection in mice deficient of CD28, we determined cytokine production by ELISA after *in vitro* restimulation of total MST or lung cells cultured in media or in the presence of 20 µg/ml α-CD3. Little or no cytokine production was detected from total MST (Fig. 2E) or lung (Fig. S2A) cells stimulated in media across all groups. However, production of IL-4, IL-13 and IL-10 by total MST (Fig. 2E) or lung (Fig. S2A) cells stimulated with α-CD3 was significantly reduced in infected CD28^−/−^ mice and infected tamoxifen treated CD28^−/lox^Cre^+/−^ mice compared to infected wild-type mice. Impaired Th2 cytokine production was further confirmed by intracellular flow cytometry, with infected CD28 deficient mice exhibiting reduced total number of CD4^+^ T cells producing Th1 and Th2 cytokines after restimulation of total MST (Fig. 2F) or lung (Fig. S2B) cells with PMA/Ionomycin *in vitro*. Furthermore, secretion of IFN-γ was abrogated in infected tamoxifen-treated CD28^−/lox^Cre^+/−^ mice compared to infected C57BL/6 mice, while it was similar between CD28^−/−^ mice and wild-type mice (Fig. 2E). In contrast, α-CD3 stimulated lung cells from infected CD28^−/−^ mice and infected tamoxifen treated CD28^−/lox^Cre^+/−^ mice had significantly increased IFN-γ production compared to infected C57BL/6 mice (Fig. S2A). Total MST (Fig. S3) or lung (Fig. S2C) cells from naive mice cultured in media or in the presence α-CD3 produced no detectable cytokines except for IFN-γ that was detected in all mice strains after stimulating cells with α-CD3. Taken together, these data suggest that abrogating CD28 costimulation impairs production of host protective Th2 cytokines during *N. brasiliensis* secondary infection.

### Development of T_FH_ cells and memory CD4^+^ T cells requires CD28 during secondary infection with *N. brasiliensis*

**Figure 3:**
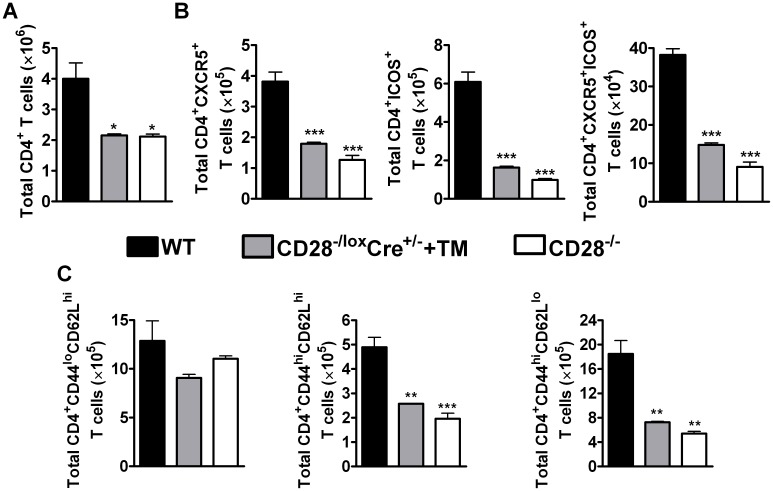
CD28 is necessary for development of T_FH_ cells and optimal activation of CD4^+^ T cells. Single cell suspension was prepared from mediastinal lymph node and cells were stained for flow cytometric analysis. (**A**), Absolute numbers of CD4^+^ T cells in the lymph node. (**B**), Absolute numbers of CD4^+^CXCR5^+^, CD4^+^CXCR5^+^ICOS^+^ and CD4^+^ICOS^+^ T cells recruited to the mediastinal lymph node. (**C**) Total number of T cell subsets infiltrating the draining lymph node. T cells subsets were differentiated based on the following markers: naive (CD4^+^CD44^lo^CD62L^hi^), central memory (CD4^+^CD44^hi^CD62L^hi^) and effector memory (CD4^+^CD44^hi^CD62L^lo^) T cells. Data represents two independent experiments. n = 5–6 mice per experiment. *P<0.05, **P<0.01, and ***P<0.001 vs C57BL/6 mice using One-Way ANOVA with Bonferroni's post test.

To further investigate possible cellular mechanisms, CD4^+^ T cell subsets were compared at 5 days post secondary infection. The absolute number of CD4^+^ T cells was significantly reduced in infected tamoxifen-treated CD28^−/lox^Cre^+/−^ mice compared to infected wild-type mice (Fig. 3A) and untreated CD28^−/lox^Cre^+/−^ littermate control (Fig. S1D). Similarly, infected CD28^−/−^ mice had reduced numbers of CD4^+^ T cells compared to infected C57BL/6 mice (Fig. 3A), while there was no difference in the number of CD4^+^ T cells between infected CD28^−/−^ mice and infected untreated CD28^−/lox^Cre^+/−^ littermate control mice (Fig. S1D). Furthermore, the absolute number of T follicular helper cells (T_FH_) expressing CXCR5 and ICOS was significantly reduced in infected CD28^−/lox^Cre^+/−^ mice that had CD28 deleted by oral administration of tamoxifen prior to secondary *N. brasiliensis* challenge and infected CD28^−/−^ compared to infected wild-type mice (Fig. 3B). Although naive untreated CD28^−/lox^Cre^+/−^ mice had significantly increased number of CD4^+^ T cells, the number of T_FH_ cells expressing CXCR5 and ICOS was similar to naive C57BL/6 mice (Fig. S4A and B). The absolute number of CD4^+^ T cells and T_FH_ cells was significantly reduced in naive CD28^−/−^ mice compared to both naive untreated CD28^−/lox^Cre^+/−^ mice and naive wild-type C57BL/6 mice, suggesting that the absence of CD28 during thymic differentiation of CD4^+^ T cells hampers expansion of T_FH_ cells (Fig. S4A and B). These data demonstrate that the development of T_FH_ during recall responses to *N. brasiliensis* infection is critically reliant on CD28 costimulation during secondary infection.

There is evidence suggesting that memory T cells develop directly from effector CD4^+^ T cells [50]. Memory T cells are defined by the expression of lymph node homing receptor L-selectin (CD62L) [51], [52], CD44 and CD45RB in mice [53]. Flow cytometric analysis of memory T cells was conducted to investigate the development of memory T cells during recall responses to *N. brasiliensis* secondary infection. The total number of naive CD4^+^ T cells (CD4^+^CD62L^hi^CD44^lo^) was similar amongst all infected groups of mice (Fig. 3C). Interestingly, interfering with CD28 costimulation prior to secondary infection with *N. brasiliensis* in infected tamoxifen-treated CD28^−/lox^Cre^+/−^ mice had a profound impact on the recruitment and/or expansion of central memory (CD4^+^CD62L^hi^CD44^hi^) and effector memory (CD4^+^CD62L^lo^CD44^hi^) T cells, as indicated by significantly reduced numbers compared to infected C57BL/6 mice (Fig. 3C). Infected CD28^−/−^ mice had reduced numbers of central and effector memory CD4^+^ T cells compared to infected C57BL/6 mice (Fig. 3C). Importantly, there was no difference in the number of naive and effector memory CD4^+^ T cells between naive C57BL/6 mice and naive untreated CD28^−/lox^Cre^+/−^ littermate control mice, except that naive littermate control mice had significantly more central memory CD4^+^ T cells than naive C57BL/6 mice (Fig. S4C). Naive CD28^−/−^ mice had reduced numbers of naive, central and effector memory CD4^+^ T cells compared to naive C57BL/6 mice and naive untreated CD28^−/lox^Cre^+/−^ mice (Fig. S4C). Together, these data suggest that CD28 is essential for the expansion of central and effector memory CD4^+^ T cells during re-infection with *N. brasiliensis*.

### CD28 influences B cell development in the draining lymph node during secondary infection with *N. brasiliensis*

**Figure 4:**
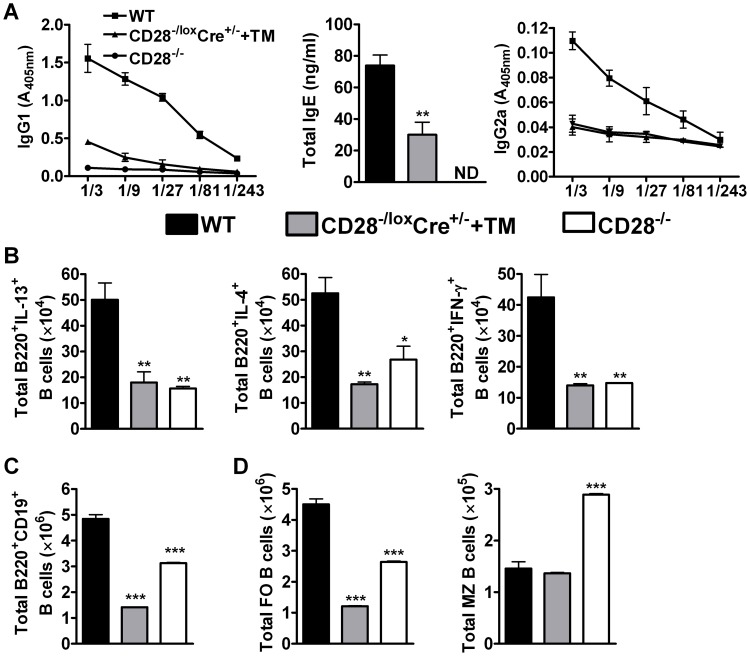
B cell development in the mediastinal lymph node is affected by CD28 deletion on CD4^+^ T cells. Blood was collected from reinfected mice by cardiac puncture and centrifuged at 8×g for 10 min in serum separator tubes. (**A**), Serum antibody titres of *N. brasiliensis* specific IgG1, IgG2a and total IgE were determined by ELISA. Single cells suspension was prepared from mediastinal lymph nodes and cells stained for flow cytometric analysis. (**B**), Total number of cytokine producing B220^+^ B cells was determined by intracellular flow cytometric analysis after re-stimulation of total MST cells with 50 ng/ml PMA, 250 ng/ml ionomycin and 200 µM monensin for 4 h at 37°C. (**C**) Total numbers of CD19^+^B220^+^ B cells population recruited into the mediastinal lymph node. (**D**)Absolute numbers of different B cell subsets recruited into the draining mediastinal lymph node. B cell subsets were differentiated based on the following markers: follicular B cells (FO, CD19^+^B220^+^CD21^hi^CD23^hi^) and marginal zone B cells (MZ, CD19^+^B220^+^CD21^hi^CD23^lo^). Data represents two independent experiments. n = 5–6 mice per group. *P<0.05 and **P<0.01 vs C75BL/6 using One-Way ANOVA with Bonferroni post test.

To investigate whether impairing CD28 signalling on CD4^+^ T cells alters B cell function, we determined serum antibody titers by ELISA. Interestingly, infected CD28^−/−^ mice and infected tamoxifen-treated CD28^−/lox^Cre^+/−^ mice had significantly reduced antigen-specific type 1 and type 2 antibody isotypes including total IgE compared to infected wild-type mice (Fig. 4A), or infected untreated CD28^−/lox^Cre^+/−^ littermate control (Fig. S1C). This data suggests that mice deficient of CD28 costimulation have impaired humoral immunity. Moreover, we determined the ability of B220^+^ B cells to produce cytokines by stimulating total MST cells with PMA/Ionomycin and detecting cytokine secretion by intracellular flow cytometric analysis. The absolute number of B220^+^ B cells secreting IL-4, IL-13 and IFN-γ were significantly reduced in both infected tamoxifen treated CD28^−/lox^Cre^+/−^ mice and infected CD28^−/−^ mice compared to infected wild-type mice (Fig. 4B). These data suggest that interfering with CD28 costimulation on CD4^+^ T cells impacts on the ability of B cells to produce antibodies and cytokines during *N. brasiliensis* infection.

We further analysed the development of B cell subsets in the MST of infected mice by flow cytometry. Infected CD28^−/−^ mice had a reduced number of CD19^+^B220^+^ B cells (Fig. 4C) and follicular B cells (Fig. 4D) while the number of marginal zone B cells (Fig. 4D) was doubled compared to infected wild-type mice. There was no difference in the number of CD19^+^B220^+^ B cells between infected CD28^−/−^ and infected CD28^−/lox^Cre^+/−^ littermate mice (Fig. S1G), although the number of follicular and non-follicular B cells was significantly reduced in infected CD28^−/−^ mice compared to infected littermate control mice (Fig. S1G). Infected CD28^−/lox^Cre^+/−^ mice given tamoxifen prior to secondary infection had a reduced number of CD19^+^B220^+^ B cells (Fig. 4C) due to strikingly reduced numbers of follicular B cells compared to infected wild-type mice (Fig. 4D) or infected untreated CD28^−/lox^Cre^+/−^ littermate control mice (Fig. S1G). In contrast, the absolute number of marginal zone B cells was similar between infected tamoxifen treated CD28^−/lox^Cre^+/−^ mice and infected wild-type mice (Fig. 4D). The similarly reduced numbers of follicular B cells in infected CD28^−/−^ mice and infected tamoxifen-treated CD28^−/lox^Cre^+/−^ mice, suggests that the expansion and/or recruitment of follicular B cells requires interaction between B cells and CD4^+^ T cells responsive to CD28 costimulation during secondary infection. Interestingly, naive C57BL/6 mice and naive untreated CD28^−/lox^Cre^+/−^ mice had similar numbers of CD19^+^B220^+^ B cells (S5A), follicular B cells (Fig. S5B) and marginal zone B cells (Fig. S5B). In contrast, naive CD28^−/−^ had significantly reduced number of CD19^+^B220^+^ B cells (Fig. S5A), follicular B cells (Fig. S5B) and an increased number of marginal zone B cells (Fig. S5B) compared to both naive C57BL/6 mice and naive untreated CD28^−/lox^Cre^+/−^ mice. Together, these data suggest that cellular interactions between CD4^+^ T cells expressing CD28 and CD19^+^B220^+^ B cells are required for the expansion and/or recruitment of follicular B cells into the lymphoid tissue during *N. brasiliensis* secondary infection.

## Discussion

The contribution of CD28 costimulation during recall of memory responses to infections has remained controversial despite numerous attempts to study it. Some studies have suggested that CD28 costimulation is not required for recall of protective memory responses to nematode infections [8], [18]. Furthermore, infection of CD28^−/−^ mice with the fungi *B. dermatitidis* showed that development of memory responses is CD28 independent [19]. In contrast, the absence of CD28 costimulation during recall of memory responses to *T. gondii* infection [23] and viral infections [24], [25] inhibited the development of protective memory responses. The controversy surrounding the requirement of CD28 for recall of protective memory responses may depend on the pathogen itself. To address these questions for nematode infections, we utilised CD28^−/−^ mice, and more importantly a recently established conditional CD28 deleting mouse strain (CD28^−/lox^Cre^+/−^), where CD28 deletion is induced by oral administration of the estrogen analogue tamoxifen [47]. The latter mouse model allowed for abrogation of CD28 costimulation after primary infection with *N. brasiliensis*, ensuring that priming of the immune response occurred in the presence of CD28 costimulation.

Primary infection in CD28^−/−^ mice with *N. brasiliensis* showed that CD28 costimulation is essential for development of optimal T cell immunity, as CD28^−/−^ mice had delayed adult worm expulsion in the small intestines. This was associated with impaired IL-13 production, known to play a crucial role in driving worm expulsion during *N. brasiliensis* infection [28], [33]. Blocking CD28 costimulation in infected wild-type mice using a novel mouse anti-mouse CD28 (E18) monoclonal antibody impaired expulsion of the worms during primary *N. brasiliensis* infection and hampered optimal development of Th2 cytokine responses (our unpublished data). This has been further confirmed by other studies that have demonstrated that interfering with CD28 costimulation during primary infection with nematodes impairs optimal development of Th2 cytokine responses [8], [18]. Interestingly, analysis of the antibody responses showed reduced titres of both type 1 and type 2 antibody isotypes, indicating a general abrogation of humoral immunity in the absence of CD28 costimulation. In contrast, CD28^−/−^ mice inoculated with *H. polygyrus* primary infection mounted a sufficient humoral immune response [10]. Hence, the requirement of CD28 costimulation in driving development of humoral immunity seems to depend on parasites causing the infection. In fact, infection with *H. polygyrus* seems to be sufficient to induce polyclonal antibody responses even in the absence of CD28 costimulation, supporting the suggestion that parasites can trigger polyclonal B cells responses [54].

Abrogating CD28 costimulation only during secondary infection in inducible CD28 deficient mice led to failure in mounting a protective memory response, strongly suggesting that CD28 costimulation is needed for efficient recall of protective immunity to *N. brasiliensis*. This conclusion is in contrast with previous findings using CTLA4-Ig to block the CD28 ligands CD80 and CD86 [8]. A possible explanation is that CTLA4-Ig exerts additional effects which may confound the situation. Furthermore, this fusion protein also prevents ligation of endogenous CTLA-4 expressed by regulatory and activated T-cells, which may partially counterbalance the desired effect. Failure to recall protective memory responses in inducible CD28 deficient mice was accompanied by strikingly reduced production of Th2 cytokines, particularly IL-4 and IL-13 by total MST or lung cells as well as impaired cytokine production by CD4^+^ T cells and B220^+^ B cells. These results are in agreement with our current knowledge that CD28 costimulation enhances IL-4 receptor sensitivity and subsequently Th2 CD4^+^ T cell differentiation [55], [56], the latter being involved in mediating protective immunity against re-infection with *N. brasiliensis* [38] by lung-resident CD4^+^ T cells. This was further confirmed by a recent study from our laboratory that demonstrated the importance of lung-resident CD4^+^ T cells in driving recall of protective immunity to *Nippostrongylus brasiliensis* re-infection after blocking T cells migration into the lungs with Fingolimod (FTY720) [39].

Previous studies have suggested a number of pathways governing the development of memory CD4^+^ T cells. In a study by Hu and colleagues, memory CD4^+^ T cells were shown to develop directly from effector CD4^+^ T cells that reverted to a resting state, suggesting a linear pathway for memory T cells generation [50]. However, other studies have suggested a more complex pathway for central memory T cell generation comprised of heterogeneous memory T cell populations [57]. In our study, the absolute number of CD4^+^ T cells was reduced in infected CD28^−/lox^Cre^+/−^ mice that had CD28 deleted prior to secondary *N. brasiliensis* challenge and infected CD28^−/−^ mice compared to infected C57BL/6 mice. These data imply that CD28 co-stimulation is important for expanding the CD4^+^ T cell compartment in the lymphoid tissue. Furthermore, the total number of central and effector memory CD4^+^ T cells was markedly reduced in the absence of CD28 costimulation during secondary *N. brasiliensis* infection, regardless of whether CD28 was constitutively missing or deleted after priming. Moreover, naive CD28^−/−^ mice had a reduced number of CD4^+^ T cells compared to naive C57BL/6 mice and naive untreated CD28^−/lox^Cre^+/−^ littermate control mice. Interestingly, naive untreated CD28^−/lox^Cre^+/−^ mice and naive CD57BL/6 mice showed similar numbers of CD4^+^ T cell subsets except for an increased number of CD4^+^ T cells and central memory cells in littermate control mice, demonstrating that untreated CD28^−/lox^Cre^+/−^ littermate control mice are phenotypically similar to wild-type mouse until *cd28* gene deletion is induced with tamoxifen prior to re-infection. These findings suggest that CD28 costimulation is required for the expansion of the CD4^+^ T cell compartment during thymic differentiation. Furthermore, it demonstrates the importance of CD28 in the expansion of memory CD4^+^ T cells during *N. brasiliensis* re-infection.

Interruption of CD28 costimulation also affected humoral immunity, as shown by reduced serum titres of type 1 and type 2 antibody isotypes in infected CD28^−/−^ mice and infected tamoxifen-treated CD28^−/lox^Cre^+/−^ mice compared to infected C57BL/6 mice. This data concurred with a previous study, that found reduced total IgE titres in sera from mice treated with CTLA4-Ig during re-infection with *N. brasiliensis* [8]. In contrast, serum levels of antigen-specific IgG1 and IgE were increased in CD28^−/−^ mice during *H. polygyrus* secondary challenge [18]. Therefore, the importance of CD28 costimulation in sustaining CD4^+^ T cells dependent memory antibody responses seems to be differentially affected by parasite causing the infection.

In a study by Zaretsky and colleagues, IL-4 producing Th2 cells were shown to possess the capacity to differentiate into T_FH_ cells during immunisation with *S. mansoni* antigens [58]. This was further confirmed in a study by King and Mohrs that demonstrated that in a Th2 setting induced by infection with *H. polygyrus*, the majority of IL-4 producing CD4^+^ T cells in the reactive lymph nodes co-express canonical T_FH_ cells markers and localised within the B cell follicles [59]. Our data showed that in the absence of CD28 costimulation, the expansion of T_FH_ cells expressing the canonical markers CXCR5 and ICOS were reduced during re-infection with *N. brasiliensis*. ICOS has been shown to play an essential role in maintaining the expression of CXCR5 on CD4^+^ T cells during SRBC immunisation and enhances GC formation and antibody production [60]. Hence, we concluded that CD28 costimulation is important for development of T_FH_ cells in the reactive lymph nodes during re-infection with the nematode *N. brasiliensis*. Together, these findings strongly demonstrate an important role played by CD28 costimulation during recall of protective memory responses to *N. brasiliensis* infection. CD28 costimulation seems to be required throughout the infection period to sustain the development of protective memory responses. These findings are in stark contrast to the normal development of protective memory responses exhibited by CD28^−/−^ mice infected with *H. polygyrus* [18]. A recent study by Harvie and colleagues showed that the lungs are a crucial site harbouring protective immunity against *N. brasiliensis* re-infection [38]. The differences observed in the requirement of CD28 costimulation during recall of memory responses to *N. brasiliensis* and *H. polygyrus* may be due to different migration patterns of the parasites within the host. *H. polygyrus* is a completely enteric parasite while *N. brasiliensis* migrates via the lungs to the intestines.

The mechanisms involved in recall of protective immunity to *N. brasiliensis* re-infection seem to involve both the innate and adaptive arm of the immune response. Voehringer and colleagues showed that mice deficient in eosinophils failed to expel *N. brasiliensis* adult worms at day 7 after secondary exposure despite the presence of Th2 cells in the lungs [36]. This was further corroborated by a subsequent study by Knott and colleagues, showing that eosinophils contribute to host resistance to *N. brasiliensis* secondary infection by limiting the number of worms reaching the lungs early during re-infection [35]. In contrast, it was shown that eosinophils in the gut are not required for worm expulsion in either primary or secondary *N. brasiliensis* infection [35]. Alternatively activated macrophages do not play a role in the expulsion of *N. brasiliensis* adult worms during primary infection in LysM^cre^IL-4Rα^−/lox^ mice [61]. However, a recent study has shown that alternatively activated macrophages are involved in the killing of *Strongloides stercoralis in vivo* and this killing effect involves a collaboration with neutrophils and complement *in vitro* [62]. Nuocytes have been shown to mediate type 2 immunity during *N. brasiliensis* infection by producing IL-13 early during infection [63]; thus, it would be interesting to determine their contributions to protective immunity during secondary infection.

The interactions between CXCR5^+^ T_FH_ cells and follicular B cells are crucial for germinal center formation in the lymphoid tissues, an important site for the development of memory B cells and antibody producing plasma cells [64], [65]. In our study, infected CD28^−/−^ mice and infected tamoxifen treated CD28^−/lox^Cre^+/−^ mice had significantly reduced absolute numbers of follicular B cells compared to infected C57BL/6 control mice. In contrast, infected CD28^−/−^ mice had increased number of marginal zone B cells while there was no difference in the number of marginal zone B cells between infected C57BL/6 mice and infected tamoxifen treated CD28^−/lox^Cre^+/−^ mice. Impaired expansion and/or recruitment of follicular B cells in infected CD28^−/−^ mice and infected tamoxifen treated CD28^−/lox^Cre^+/−^ mice might provide a plausible explanation for abrogated humoral immunity after both primary and secondary *N. brasiliensis* infection. Therefore, interfering with CD28 costimulation appears to alter the ability of CD4^+^ T cells to provide cognate help to B cells necessary for optimal antibody production and isotype class-switching.

In conclusion, our study demonstrates the essential role played by CD28 costimulation during recall of protective memory responses to *N. brasiliensis* infection. CD28 costimulation was shown to confer protection against primary infection with *N. brasiliensis* using CD28^−/−^ mice. Failure to expel adult *N. brasiliensis* worms during secondary infection was associated with diminished Th2 cytokine responses and abrogated humoral immunity particularly the production of IgG1 and total IgE. Importantly, the deficiency of CD28 costimulation impaired recruitment of memory CD4^+^ T cell sub-populations and expansion of T follicular helper cells crucial for providing help to follicular B cells.

## Materials and Methods

### Mice

C57/BL6 background CD28^−/−^ and CD28^−/lox^, rosa (CreER^T2^) (CD28^lox/−^Cre^+/−^) mice were obtained from Prof. T. Hünig at the University of Würzburg, Germany. The mice were bred and maintained in specific pathogen-free barrier conditions in individually ventilated cages at the University of Cape Town Animal Facility. All experimental mice were age and sex matched and used between 8–12 weeks of age.

### Ethics statement

This study was conducted under strict recommendation of the South African national guidelines and of the University of Cape Town practice for laboratory animal procedures. All mouse experiments were carried out in accordance to protocols approved by the Animals Research Ethics Committee of the Health Sciences Faculty, University of Cape Town (Project Number: 011/008). Care was taken to minimize suffering of the animals.

### *N. brasiliensis* infection

#### Primary infection

Mice were injected subcutaneously with 500 *N. brasiliensis* L3 suspended in 0.65–0.9% NaCl using 21-G needle (Braun, Melsungen, Germany). Mice were killed 9 and 12 days post-infection and adults worms were enumerated using a previously described method [27].

#### Secondary infection

Mice were initially injected with 500 *N. brasiliensis* L3, orally treated with 10 mg/ml Ivermectin in drinking water at seven days post-infection and shelved for 21 days prior to a secondary subcutaneous infection with 500 *N. brasiliensis* L3. Mice were killed 5 days post secondary infection by halothane inhalation and exsanguination.

### *N. brasiliensis* intestinal worm counts

Small intestines were removed from infected mice; the lumen was exposed by dissection and suspended in 0.65% NaCl. The intestines were incubated at 37°C for 4 h to allow for migration of the worms out of the lumen after which they were enumerated under a dissecting microscope (Nikon Eclipse).

### Histology

Tissue sample were fixed in buffered 4% (v/v) formaldehyde, embedded in paraffin wax and cut into 5 µm sections. The sections were stained with periodic acid-Schiff reagent (PAS) in order to visualize mucus producing goblet cells. The sections were analysed under a light microscope.

### Determination of antibody titres

*N. brasiliensis* antigen-specific serum antibody isotypes and total IgE titres from infected mice were determined as previously described [66]. Briefly, blood was collected in serum separator tubes (BD Bioscience, San Diego, CA) and centrifuged at 8 000×g for 10 min at 4°C to separate serum. The flat-bottom 96-well plates were coated with 10 µg/ml somatic *N. brasiliensis* antigen (NAg), blocked with 2% (w/v) milk powder for 2 h at 37°C and samples were loaded and incubated overnight at 4°C. Alkaline phosphatase labelled secondary antibody was added and incubated for 2 h at 37°C. The plates were developed by addition of 4-nitrophenyl substrate (Sigma). The absorbance was read at 405 nm using VersaMax microplate spectrophotometer (Molecular Devices, Germany).

### *Ex vivo* restimulation of cells and cytokines detection

Single cell suspensions were prepared by pressing the draining lymph nodes through 70 µM cell-strainers. Cells were resuspended in complete IMDM (Gibco) supplemented with 10% FCS (Gibco) and penicillin and streptomycin (100 U/ml and 100 µg/ml, Gibco). The cells were cultured at 1×10^6^ cells/ml in 96-well plates coated with α-CD3 (20 µg/ml) and incubated at 37°C in a humidified atmosphere containing 5% CO_2_. Supernatants were collected after 72 h and cytokines were measured by ELISA. Quantities of IL-4, IL-10, IFN-γ and IL-13 were measured by sandwich ELISA as previously described [66].

### Inducible deletion in conditional knock-out mice

CD28^−/lox^Cre^+/−^ mice were given 2.5 mg Tamoxifen (Sigma, Deisenhofen, Germany) in vegetable oil for four consecutive days by forced feeding.

### Flow cytometry

The following antibodies comprising the B cells antibody panel were used: B220-V500 (RA36B2), CD19-PerCP Cy5.5 (ID3), CD23-PE (B3B4), CD21-APC (7G6), CD24-PECy7 (M1/69), CD80-V450 (19-10A1), MHCII-FITC (2G9) and IgM-Biotin (RMM-1) (BD Bioscience, Erembodegem, Belgium). T cells panel consisted of the following antibodies: CD4-PerCP (RM4-5), CD62L-V500 (MEL-14), CD44-FITC (IM7), CD28-PE (37.51), CXCR5-V450 (2G8) and CD278-Biotin (7E.17G9) (BD Bioscience, Erembodegem, Belgium). Cells were acquired on a FACS Fortessa machine (BD Immunocytometry system, San Jose, CA, USA) and data was analyzed using Flowjo software (Treestar, Ashland, OR, USA).

### Intracellular flow cytometry

For detection of intracellular cytokines, MST or lung cells from *N. brasiliensis* infected mice were plated at 2×10^6^ cells/well and stimulated at 37°C for 4 h with 50 ng/ml phorbol (myristate acetate), 250 ng/ml ionomycin and 200 µM monensin in complete IMDM (all purchased from Sigma-Aldrich). Cells were stained with extracellular markers [CD4 PercP Cy5.5 (RM4-5) and B220 FITC (RA3-6B2)], fixed for 30 min on ice in 2% (w/v) paraformaldehyde and permeabilised with 0.5% saponin buffer and stained with PE-labelled anti-mouse IL-4, IL-13, and IFN-γ for 30 min. Acquisition was performed using a FACSCalibur (BD Immunocytometry Systems, San Jose, CA, USA) and data were analysed using FlowJo software (Treestar, Ashland, OR, USA).

### Statistics

Statistical analysis was conducted using GraphPad Prism 4 software (http://www.prism-software.com). Data were calculated as mean ± SD. Statistical significant was determined using the unpaired Student's *t* test, One-Way or Two-Way ANOVA with Bonferroni's post test, defining differences to C57BL/6 or untreated CD28^lox−^Cre^+/−^ as significant (*, *p*≤0.05; **, *p*≤0.01; ***, *p*≤0.001).

## Supporting Information

Figure S1 **CD28 is required for recall of memory responses during** ***N. brasiliensis*** **secondary infection.** Untreated CD28^−/lox^Cre^+/−^ littermate control, tamoxifen treated CD28^−/lox^Cre^+/−^ and CD28^−/−^ mice were re-infected with 500 L3 *N. brasiliensis* and killed 5 days post-infection. (**A**), Histogram showing CD28 expression by CD4^+^ T cells from untreated CD28^−/lox^Cre^+/−^ mice, tamoxifen treated CD28^−/lox^Cre^+/−^ mice and CD28^−/−^ mice. (**B**), Intestinal worm burdens were quantified. (**C**), Serum antibody titres of *N. brasiliensis* specific IgG1 and total IgE were determined by ELISA. Single cell suspension was prepared from mediastinal lymph node and cells were stained for flow cytometric analysis. (**D**), Absolute numbers of CD3^+^CD4^+^ T cells in the lymph node. (**E**) Total number of CD3^+^CD4^+^CXCR5^+^ and CD3^+^CD4^+^ICOS^+^ T cells recruited to the mediastinal lymph node. (**F**) Total number of T cell subsets infiltrating the draining lymph node. T cells subsets were differentiated based on the following markers: naive (CD3^+^CD4^+^CD44^lo^CD62L^hi^), effector memory (CD3^+^CD4^+^CD44^hi^CD62L^lo^) and central memory (CD3^+^CD4^+^CD44^hi^CD62L^hi^) T cells. (**G**), Total numbers of CD19^+^B220^+^ B cells, follicular B cells (FO, CD19^+^B220^+^CD21^hi^CD23^hi^), marginal zone B cells (MZ, CD19^+^B220^+^CD21^hi^CD23^lo^) and non-follicular B cells (NF, CD19^+^B220^+^CD21^lo^CD23^lo^) draining into the MST. Data is representative of three independent experiments. n = 4–6 mice per group. *P<0.05, **P<0.01, and ***P<0.001 vs CD28^−/lox^Cre^+/−^ mice given oil using One-Way ANOVA with Bonferroni's post test.
(TIF)

Figure S2 **Reduced production of protective Th2 cytokines by lung resident cells from CD28 deficient mice.** Re-infected wild-type (C57BL/6), tamoxifen treated CD28^−/lox^Cre^+/−^ and CD28^−/−^ mice were killed 5 days post-infection and lungs were collected. Single cell suspensions were prepared by digesting lung tissue with 50 U Type 1 collagenase for 1 h at 37°C and cells were re-stimulate in media or in the presence of 20 µg/ml α-CD3 for 72 h at 37°C. (**A**), Cytokine production by infected total lung cells was determined by ELISA. (**B**), Total number of CD4^+^ T cells producing cytokines was determine by intracellular flow cytometry after re-stimulating total lung cells with 50 ng/ml PMA, 250 ng/ml ionomycin and 200 µM monensin for 4 h at 37°C. (**C**), Cytokine production by naive total lung cells was determined by ELISA. Data are representative of two independent experiments. n = 5–6 mice per group. *P<0.05, **P<0.01 and ***P<0.001 vs C57BL/6 mice using One-Way or Two-Way ANOVA with Bonferroni's post test.
(TIF)

Figure S3 **Minimal cytokine production by cells from naive mouse strains.** Naive wild-type (C57BL/6), untreated CD28^−/lox^Cre^+/−^ and CD28^−/−^ mice were killed and MST harvested and pooled. Single cell suspensions were prepared from MST and cells were re-stimulated *in vitro*. Cytokine production by total mediastinal lymph node cell re-stimulated in media or in the presence of 20 µg/ml α-CD3 was determined by ELISA. Data represents two independent experiments. n = 5–6 mice per group. **P<0.01 vs C57BL/6 mice using Two-Way ANOVA with Bonferroni's post test.
(TIF)

Figure S4 **Reduced numbers of CD4^+^ T cells and effector memory CD4^+^ T cells in CD28^−/−^ mice.** Naive wild-type (C57BL/6), untreated CD28^−/lox^Cre^+/−^ and CD28^−/−^ mice were killed and mediastinal lymph nodes harvested and pooled. Single cell suspensions were prepared from pooled MST and cells were stained for flow cytometric analysis. (**A**), Total number of CD4^+^ T cells found in the MST. (**B**), Total number of CD4^+^CXCR5^+^, CD4^+^CXCR5^+^ICOS^+^ and CD4^+^ICOS^+^ T cells in the lung draining lymph node. (**C**), Total number of naive (CD4^+^CD62L^hi^CD44^lo^), central memory (CD4^+^CD62L^hi^CD44^hi^) and effector memory (CD4^+^CD62L^lo^CD44^hi^) T cells. Data are representative of two independent experiments. n = 6 mice per group. ***P<0.001 vs C57BL/6 and untreated CD28^−/lox^Cre^+/−^ mice using One-Way ANOVA with Bonferroni's post test.
(TIF)

Figure S5 **Unaltered numbers of B cell subsets in naive CD28 sufficient mice.** Naive wild-type (C57BL/6), untreated CD28^−/lox^Cre^+/−^ and CD28^−/−^ mice were killed and mediastinal lymph nodes harvested and pooled. Single cell suspensions were prepared from pooled MST and cells were stained for flow cytometric analysis. (**A**), Total number of CD19^+^B220^+^ B cells found in the MST. (**B**), Total number of follicular B cells and marginal zone B cells in the lung draining lymph node. Data are representative of two independent experiments. n = 6 mice per group. *P<0.05 and **P<0.01 vs C57BL/6 and untreated CD28^−/lox^Cre^+/−^ mice using One-Way ANOVA with Bonferroni's post test.
(TIF)
