# Supplementary figures and images for: Inducible Deletion of CD28 Prior to Secondary Nippostrongylus brasiliensis Infection Impairs Worm Expulsion and Recall of Protective Memory CD4+ T Cell Responses
Source: PLoS Pathog. 2014 Feb 6;10(2):e1003906. doi: 10.1371/journal.ppat.1003906 (PMC3916406; doi:10.1371/journal.ppat.1003906)

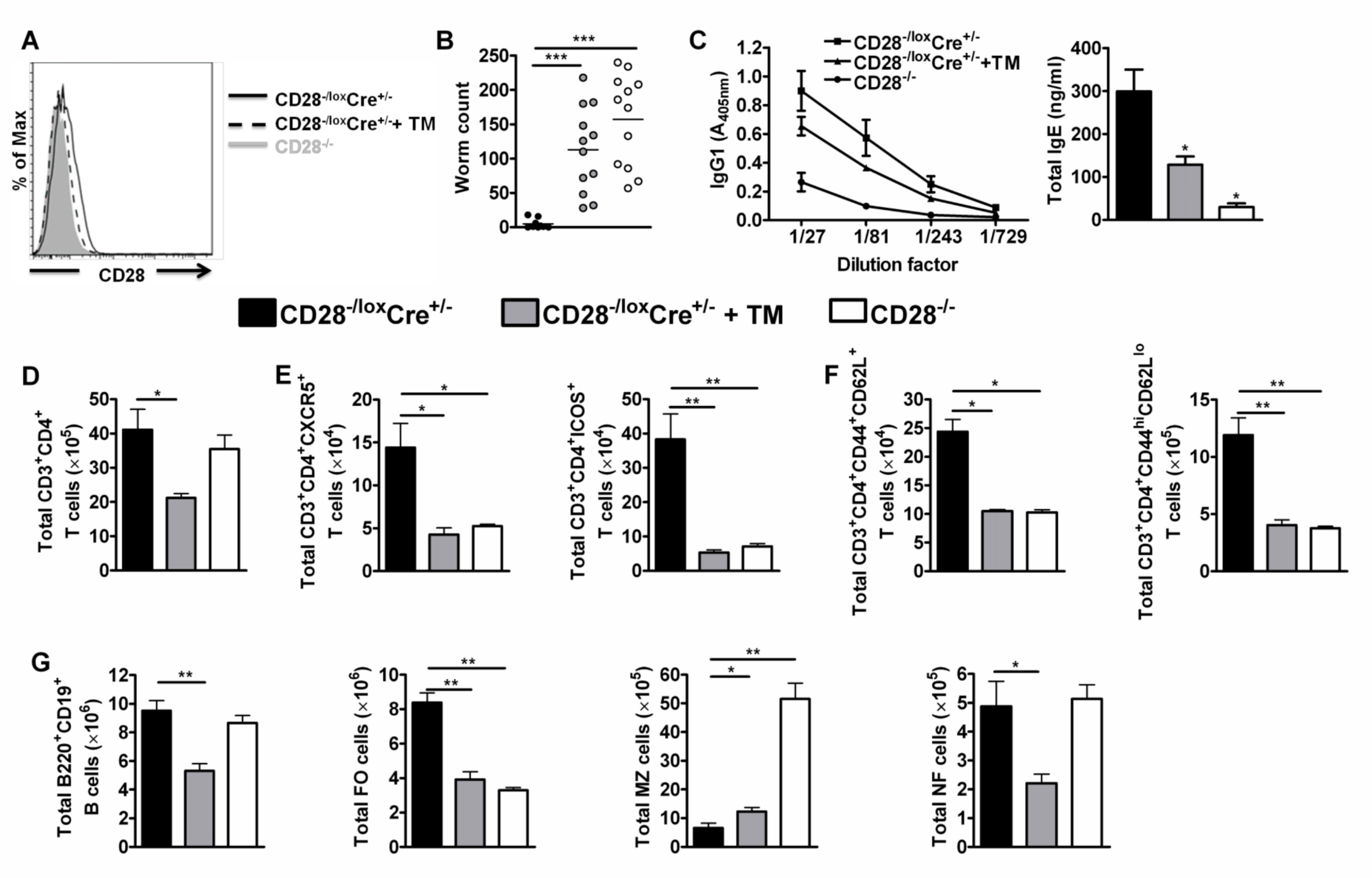

Supplement: Figure S1 — CD28 is required for recall of memory responses during N. brasiliensis secondary infection. Untreated CD28−/loxCre+/− littermate control, tamoxifen treated CD28−/loxCre+/− and CD28−/− mice were re-infected with 500 L3 N. brasiliensis and killed 5 days post-infection. (A), Histogram showing CD28 expression by CD4+ T cells from untreated CD28−/loxCre+/− mice, tamoxifen treated CD28−/loxCre+/− mice and CD28−/− mice. (B), Intestinal worm burdens were quantified. (C), Serum antibody titres of N. brasiliensis specific IgG1 and total IgE were determined by ELISA. Single cell suspension was prepared from mediastinal lymph node and cells were stained for flow cytometric analysis. (D), Absolute numbers of CD3+CD4+ T cells in the lymph node. (E) Total number of CD3+CD4+CXCR5+ and CD3+CD4+ICOS+ T cells recruited to the mediastinal lymph node. (F) Total number of T cell subsets infiltrating the draining lymph node. T cells subsets were differentiated based on the following markers: naive (CD3+CD4+CD44loCD62Lhi), effector memory (CD3+CD4+CD44hiCD62Llo) and central memory (CD3+CD4+CD44hiCD62Lhi) T cells. (G), Total numbers of CD19+B220+ B cells, follicular B cells (FO, CD19+B220+CD21hiCD23hi), marginal zone B cells (MZ, CD19+B220+CD21hiCD23lo) and non-follicular B cells (NF, CD19+B220+CD21loCD23lo) draining into the MST. Data is representative of three independent experiments. n = 4–6 mice per group. *P<0.05, **P<0.01, and ***P<0.001 vs CD28−/loxCre+/− mice given oil using One-Way ANOVA with Bonferroni's post test. (TIF) [file ppat.1003906.s001.tif]
